# Conflict of Interest Reporting by Authors Involved in Promotion of Off-Label Drug Use: An Analysis of Journal Disclosures

**DOI:** 10.1371/journal.pmed.1001280

**Published:** 2012-08-07

**Authors:** Aaron S. Kesselheim, Bo Wang, David M. Studdert, Jerry Avorn

**Affiliations:** 1 Division of Pharmacoepidemiology and Pharmacoeconomics, Department of Medicine, Brigham and Women's Hospital and Harvard Medical School, Boston, Massachusetts, United States of America; 2 Schools of Law and Population Health, University of Melbourne, Melbourne, Australia; York University, Canada

## Abstract

**Background:**

Litigation documents reveal that pharmaceutical companies have paid physicians to promote off-label uses of their products through a number of different avenues. It is unknown whether physicians and scientists who have such conflicts of interest adequately disclose such relationships in the scientific publications they author.

**Methods and Findings:**

We collected whistleblower complaints alleging illegal off-label marketing from the US Department of Justice and other publicly available sources (date range: 1996–2010). We identified physicians and scientists described in the complaints as having financial relationships with defendant manufacturers, then searched Medline for articles they authored in the subsequent three years. We assessed disclosures made in articles related to the off-label use in question, determined the frequency of adequate disclosure statements, and analyzed characteristics of the authors (specialty, author position) and articles (type, connection to off-label use, journal impact factor, citation count/year). We identified 39 conflicted individuals in whistleblower complaints. They published 404 articles related to the drugs at issue in the whistleblower complaints, only 62 (15%) of which contained an adequate disclosure statement. Most articles had no disclosure (43%) or did not mention the pharmaceutical company (40%). Adequate disclosure rates varied significantly by article type, with commentaries less likely to have adequate disclosure compared to articles reporting original studies or trials (adjusted odds ratio [OR] = 0.10, 95%CI = 0.02–0.67, *p* = 0.02). Over half of the authors (22/39, 56%) made no adequate disclosures in their articles. However, four of six authors with ≥25 articles disclosed in about one-third of articles (range: 10/36–8/25 [28%–32%]).

**Conclusions:**

One in seven authors identified in whistleblower complaints as involved in off-label marketing activities adequately disclosed their conflict of interest in subsequent journal publications. This is a much lower rate of adequate disclosure than has been identified in previous studies. The non-disclosure patterns suggest shortcomings with authors and the rigor of journal practices.

*Please see later in the article for the Editors' Summary*

## Abstract 2

Aaron Kesselheim and colleagues investigate conflict of interest disclosures in articles authored by physicians and scientists identified in whistleblower complaints alleging illegal off-label marketing by pharmaceutical companies.

## Editor's Summary

**Background:**

Off-label use of pharmaceuticals is the practice of prescribing a drug for a condition or age group, or in a dose or form of administration, that has not been specifically approved by a formal regulatory body, such as the US Food and Drug Administration (FDA). Off-label prescribing is common all over the world. In the US, although it is legal for doctors to prescribe drugs off-label and discuss such clinical uses with colleagues, it is illegal for pharmaceutical companies to directly promote off-label uses of any of their products. Revenue from off-label uses can be lucrative for drug companies and even surpass the income from approved uses. Therefore, many pharmaceutical companies have paid physicians and scientists to promote off-label use of their products as part of their marketing programs.

**Why Was This Study Done?:**

Recently, a number of pharmaceutical companies have been investigated in the US for illegal marketing programs that promote off-label uses of their products and have had to pay billions of dollars in court settlements. As part of these investigations, doctors and scientists were identified who were paid by the companies to deliver lectures and conduct other activities to support off-label uses. When the same physicians and scientists also wrote articles about these drugs for medical journals, their financial relationships would have constituted clear conflicts of interest that should have been declared alongside the journal articles. So, in this study, the researchers identified such authors, examined their publications, and assessed the adequacy of conflict of interest disclosures made in these publications.

**What Did the Researchers Do and Find?:**

The researchers used disclosed information from the US Department of Justice, media reports, and data from a non-governmental organization that tracks federal fraud actions, to find whistleblower complaints alleging illegal off-label promotion. Then they identified the doctors and scientists described in the complaints as having financial relationships with the defendant drug companies and searched Medline for articles authored by these experts in the subsequent three years. Using a four step approach, the researchers assessed the adequacy of conflict of interest disclosures made in articles relating to the off-label uses in question.

Using these methods, the researchers examined 26 complaints alleging illegal off-label promotion and identified the 91 doctors and scientists recorded as being involved in this practice. The researchers found 39 (43%) of these 91 experts had authored 404 related publications. In the complaints, these 39 experts were alleged to have engaged in 42 relationships with the relevant drug company: the most common activity was acting as a paid speaker (n = 26, 62%) but also writing reviews or articles on behalf of the company (n = 7), acting as consultants or advisory board members (n = 3), and receiving gifts/honoraria (n = 3), research support funds (n = 2), and educational support funds (n = 1). However, the researchers found that only 62 (15%) of the 404 related articles had adequate disclosures—43% (148) had no disclosure at all, 4% had statements denying any conflicts of interest, 40% had disclosures that did not mention the drug manufacturer, and 13% had disclosures that mentioned the manufacturer but inadequately conveyed the nature of the relationship between author and drug manufacturer reported in the complaint. The researchers also found that adequate disclosure rates varied significantly by article type, with commentaries significantly less likely to have adequate disclosure compared to articles reporting studies or trials.

**What Do These Findings Mean?:**

These findings show the substantial deficiencies in the adequacy of conflict-of-interest disclosures made by authors who had been paid by pharmaceutical manufacturers as part of off-label marketing activities: only one in seven authors fully disclosed their conflict of interest in their published articles. This low figure is troubling and suggests that approaches to controlling the effects of conflicts of interest that rely on author candidness are inadequate and furthermore, journal practices are not robust enough and need to be improved. In the meantime, readers have no option but to interpret conflict of interest disclosures, particularly in relation to off-label uses, with caution.

**Additional Information:**

Please access these Web sites via the online version of this summary at http://dx.doi.org/10.1371/journal.pmed.1001280.

- The US FDA provides a guide on the use of off-label drugs
- The US Agency for Healthcare Research and Quality offers a patient guide to off-label drugs
- ProPublica offers a web-based tool to identify physicians who have financial relationships with certain pharmaceutical companies
- Wikipedia has a good description of off-label drug use (note that Wikipedia is a free online encyclopedia that anyone can edit; available in several languages)
- The Institute for Medicine as a Profession maintains a list of policies regulating physicians' financial relationships that are in place at US-based academic medical centers

## Introduction

Collaborations between physicians and industry are integral to medical research. However, some professional relationships with drug and device companies may also impact the design [1] and outcomes [2] of biomedical research, and the reporting of research findings [3]. The question of how best to manage financial conflicts of interest among physicians and scientists is hotly debated. Rather than seeking to sever all ties [4], many policymakers and expert bodies have recommended full disclosure as the first step in any mitigation strategy [5]. However, this approach depends on physicians being forthcoming, which may not occur [6]–[9].

Prescribing drugs for purposes outside those approved by the US Food and Drug Administration (FDA)—“off-label” use—is common in clinical practice and may be appropriate if well-grounded in solid clinical trial findings [10]. Promotion of off-label uses is a fertile area for investigating undisclosed entanglements between researchers and industry for several reasons. First, while it is illegal for companies to directly promote off-label uses, it is legal for independent physicians to discuss such uses with their colleagues. Second, because off-label uses by definition come with no FDA guidance, and often little or no scientific foundation [11], expert opinion may have considerable sway over prescriber behavior [12]. Third, revenue from off-label use can be lucrative for drug companies, sometimes dwarfing that derived from approved uses [13]. These factors make physicians who are willing to advocate off-label prescribing a valuable commodity to pharmaceutical companies.

A growing number of companies have been investigated for engaging in illegal off-label marketing, with the total combined value of the settlements reaching billions of dollars [14]. To date, government prosecutors have generally not targeted the expert advocates the companies enlist [15]. However, the government's investigations routinely identify physicians and scientists paid by manufacturers to deliver lectures and author peer-reviewed articles that support off-label uses [16]. Using a list of physicians and scientists identified by whistleblowers in a sample of off-label prosecutions, we gathered publications authored by these advocates following their reported relationship, and assessed the adequacy of disclosures made in these publications.

## Methods

### Setting and Participants

We used complaints filed by whistleblowers in “qui tam” cases brought under the US federal False Claims Act (FCA) to identify authors who were compensated by companies allegedly involved in off-label promotion. The FCA prohibits submission of false claims to the government for reimbursement. Parties report potential violations by filing a sealed, confidential complaint, which the US Department of Justice (DOJ) investigates. If the evidence supports the allegations, the DOJ may take over the enforcement action, and the whistleblower's complaint is usually unsealed.

Using a search of DOJ press releases [17], media reports in Lexis-Nexis (Dayton, Ohio) [18], and data from Taxpayers Against Fraud, a non-governmental organization that tracks federal fraud actions, we identified 23 FCA enforcement actions from 1996 to 2010 relating to allegations of illegal off-label marketing schemes in which complaints were unsealed because the defendant settled the case or the government took a lead in the investigation (21 settled by the end of our study period). There were 48 unsealed complaints associated with these cases, which we obtained via the DOJ website, federal court filings [19], and contacting the lawyers involved.

We searched the complaints to identify physicians and scientists allegedly paid by a pharmaceutical company as part of its off-label marketing. There were 91 physicians and scientists in 26 different complaints whose names, involvement dates (range: 1999–2007), and role in the off-label promotional activities were described. We recorded their affiliation with the manufacturer and the time frame of that affiliation.

Since nearly all whistleblower cases against pharmaceutical manufacturers are eventually settled, the complaints we examined were not scrutinized in court. Thus, they remain allegations, and some whistleblower reports may have identified physicians who were not in fact recipients of payments, or may have been inaccurate in other respects. However, in all cases, the DOJ had reviewed the evidence (including the whistleblower complaints), conducted its own investigation, and determined that the case was strong enough to justify unsealing the complaint or having the DOJ join the prosecution.

### Medical Literature Search

To identify articles authored by the 91 physicians and scientists, one of us (BW) conducted Boolean searches of PubMed [20] (in June 2011) looking for matches between the person's name and one of the following: name of the defendant manufacturer; name of the drug(s) at issue in the litigation; terms describing the class of the drug (e.g., *antiepileptic*); or terms describing the therapeutic specialties in which the drug was used (e.g., *neurolog!* or *psych!*). In each search, the time window began six months after the earliest date of the author's affiliation with the manufacturer, and ended 36 months after the last mentioned date. The six-month mark allowed for publication lag (opportunity to receive and edit proofs on manuscripts already submitted, if it happened to be the author's first relationship with the pharmaceutical company); the 36-month mark corresponds to the International Committee of Medical Journal Editors' (ICJME's) standard for the time period over which a financial conflict of interest should be disclosed [21]. As a sensitivity analysis, we also limited the searches to twelve months after the final reported date.

### Assessment of Relatedness of Publications

After obtaining the full text of all articles identified through this process, two of us (ASK, BW) independently reviewed each publication to determine its relevance to the corporate relationship mentioned in the whistleblower complaint. Specifically, following Okike et al. [9], the articles were classified as “related” or “unrelated” to the drug(s) to which the author was linked in the whistleblower's complaint. We based this again on the ICMJE, which calls for disclosure of “interactions with ANY [sic] entity that could be considered broadly relevant to the work” [21]. Thus, related articles covered any use of the drug at issue [22], discussed diagnoses or diseases treated by the drug [23], and mentioned other medications in the same therapeutic class or alternative non-pharmaceutical therapies [24]. Unrelated articles addressed topics in other fields or other drug classes [25]–[26]. Whereas in Okike et al., related articles were further broken down into directly and indirectly related articles, we chose not to pursue this subdivision because articles in both categories should carry an appropriate conflict of interest disclosure, if a financial relationship exists.

Reliability testing on the relatedness assessments showed excellent agreement between the independent reviewers. Among 528 articles that underwent double review, there was 98% agreement and the kappa score was 0.95 (standard error [SE] = 0.02). Disagreements were resolved by consensus among the investigators.

### Outcomes: Assessment of Disclosures

For all related articles, we assessed adequacy of disclosures in a four-step process. First, we determined whether the printed article contained any disclosure, including formal conflict of interest statements, statements of financial support for the study, and acknowledgments (other than mere expressions of gratitude to colleagues or personal assistants). Supplemental on-line disclosures, if any, were obtained. Second, among articles with disclosures, we identified those with declarations of no conflict. Third, for the remaining articles, we determined whether the disclosure mentioned the defendant manufacturer.

Finally, we analyzed disclosure statements that mentioned the defendant manufacturer to determine whether the statement adequately matched the author's financial relationship described in the whistleblower's complaint. An adequate disclosure was defined as one in which the existence of a financial relationship between the author and defendant manufacturer, as revealed in the complaint, was also stated in the published article. An inadequate disclosure was defined as one in which a personal connection was not mentioned. Two investigators (ASK, BW) independently determined adequacy, with disagreements resolved by consensus. We also calculated inter-rater reliability on this judgment. Among 105 disclosures reviewed, there was 90% agreement and the kappa score was 0.78 (SE = 0.06), indicating good-to-excellent agreement [27]. As a companion analysis, we assessed disclosure rates among articles we categorized as “unrelated.”

### Collection of Other Information on Articles and Authors

With respect to authors, we identified their primary clinical specialty or scientific field from information available in their publications and the complaint; for physicians, specialty was confirmed through searches of on-line databases. We also categorized authors by authorship position (sole, first, middle, last). We categorized publications by type (trials/studies, reviews, commentaries/editorials/letters) and noted whether they mentioned an off-label use in the title, abstract, or conclusion.

We used the Thompson Reuters database [28] to obtain citation counts for the publications (as of July 2011) and the Journal Impact Factors at the year of publication. Each citation count was divided by the years since publication to obtain a time-adjusted measure. Journal Impact Factors were unavailable for nine publications. For an additional 28 publications, the Journal Impact Factor was not available for the relevant year so we obtained it by contacting journal offices or assigning the Journal Impact Factor from the nearest year available.

### Statistical Analysis

We calculated counts of authors and articles by the adequacy of disclosure. For each characteristic of interest, we estimated a hierarchical logistic regression model for the probability of adequate disclosure that included independent random intercepts for author and journal and fixed effects for article or journal characteristic as appropriate (R software, version 2.12.2) [29]. For example, in the analysis of author specialty, this model estimated the odds ratio of adequate disclosure comparing articles authored by specialists in surgery/urology, nephrology, neurology, or other fields to psychiatrists, the reference group, accounting for the correlated likelihood of disclosure for articles that were penned by the same author or published in the same journal.

## Results

Of 91 physicians and scientists in the complaints, 39 (43%) authored 404 related publications, 16 (18%) authored 124 unrelated publications, and 51 (56%) authored no publications in the period of interest. Fifteen of the 16 authors who published the unrelated articles also published related articles. The 39 authors, and their 404 related publications, became our study sample. These 39 authors emerged from complaints involving 18 drugs. The majority (11/18, 61%) were psychoactive compounds, including antidepressants (e.g., escitalopram [Lexapro]), antipsychotics (e.g., olanzapine [Zyprexa]), antiepileptics (e.g., oxcarbazepine [Trileptal]), and central nervous system stimulants (e.g., sodium oxybate [Xyrem]).

### Author Characteristics

The 39 authors of related articles consisted of 13 psychiatrists, nine nephrologists, seven surgeons/urologists, five neurologists, two non-clinician PhDs, one anesthesiologist, one internist, and one endocrinologist. The authors were alleged in the complaints to have engaged in 42 relationships with the pharmaceutical manufacturer (three authors had two or more different types). The most common was acting as a paid speaker (n = 26, 62%). Authors also wrote reviews or articles on behalf of the company (n = 7), acted as consultants or advisory board members (n = 3), and received gifts/honoraria (n = 3), research support funds (n = 2), and educational support funds (n = 1).

The 52 physicians and scientists who did not author any related publications allegedly engaged in 53 relationships. The most common was receiving gifts/honoraria (n = 30, 57%), followed by acting as a paid speaker (n = 21, 40%). One complaint referred to a consultant or advisory board member relationship and another complaint referred to research support.

### Characteristics of Related Articles

The median number of related articles per author was 7 (interquartile range [IQR] 2–12). These articles included 258 studies or trials with original data (64% of articles), 97 clinical reviews (24%), and 49 editorials, commentaries, or letters (12%). Sixty-two (15%) articles were sole-authored; in 87 (22%), the author appeared first and in 95 (23%) the author appeared last. The median Journal Impact Factor was 3.6 (IQR 2.0–4.8) and each article was cited a median of 2.5 times (IQR 1.0–5.5). Among the 404 related articles, 177 (44%) discussed an off-label use of the drug.

### Adequacy of Disclosure

**Figure 1:**
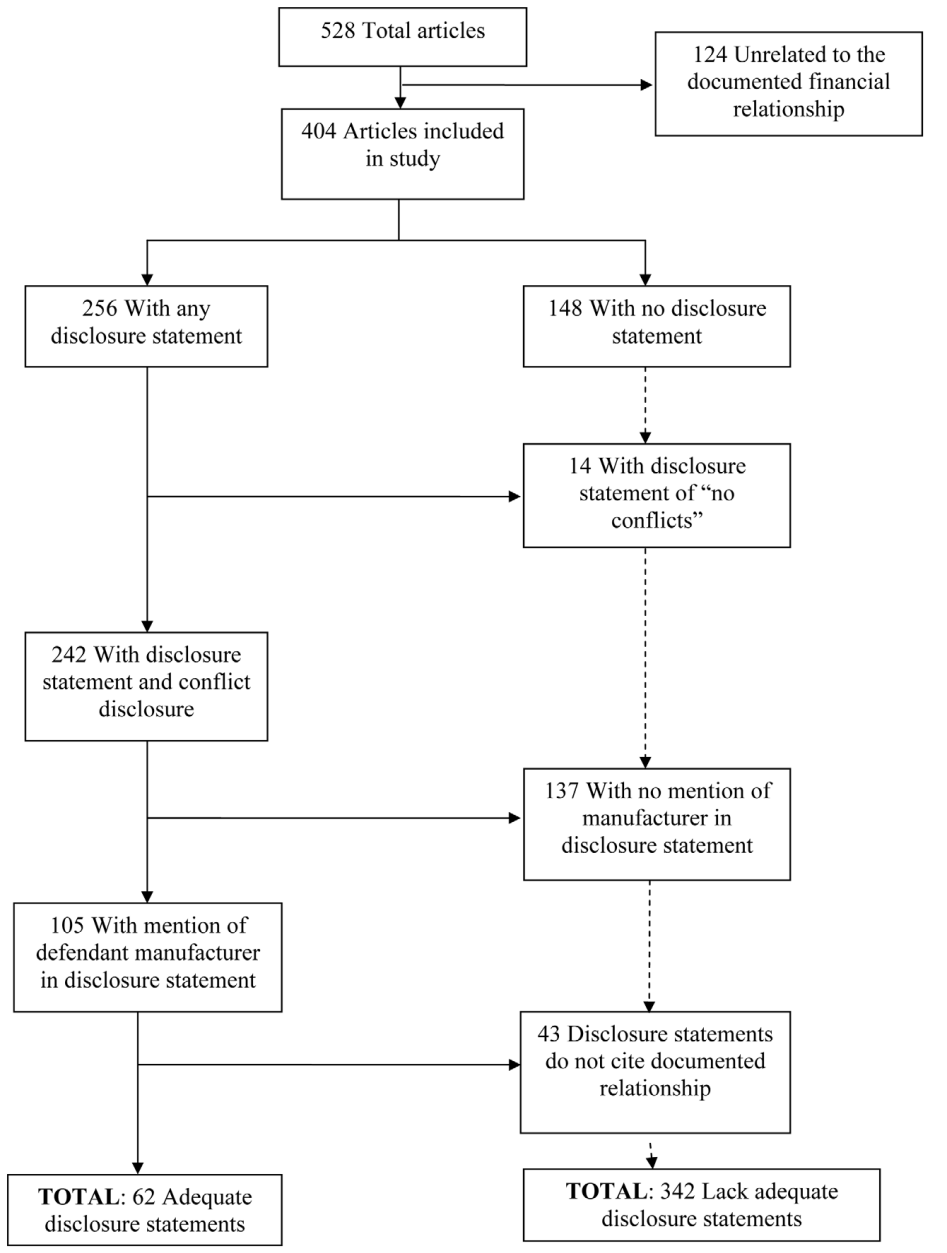
Flowchart categorization of articles in sample according to the adequacy of their conflicts of interest disclosures.

A total of 62 (15%) of the 404 related articles had adequate disclosures and 342 (85%) had inadequate disclosures (Figure 1). Among articles without adequate disclosures, 43% (148/342) had no disclosure at all, 4% had statements denying any conflicts of interest, 40% had disclosures that did not mention the manufacturer, and 13% had disclosures that mentioned the manufacturer but inadequately conveyed the nature of the relationship between author and manufacturer reported in the complaint. Table 1 presents some examples of adequate and inadequate disclosures.

**Table 1:** Examples of adequate and inadequate disclosure.

Descriptions Alleged in Qui Tam Complaints	Article Type	Disclosure in Peer-Reviewed Article
**Adequate disclosure**		
“[Author] received $94,250 in 2003 in payments from AstraZeneca for his presentations.”	Randomized trial of competitor drug in same class	“[Author] has previously been a consultant for, and on the speakers' bureaus of, AstraZeneca, Bristol-Myers Squibb, Eli Lilly, Janssen, and Pfizer Pharmaceuticals.”
“To convince doctors to prescribe Zyprexa at these extremely high dosages, Lilly also funded the [author] study.”	Consensus statement on use of drug at issue and other drugs in class	“[Author] has received honoraria and/or research support from AstraZeneca, Bristol-Myers Squibb, Glaxo, and Lilly.”
“[Author] … discussed the off-label use of Zonegran for mood stabilization and the treatment of mania.”	Retrospective chart review study of drug's use	“[Author] has received grant/research support from Elan Pharmaceutical and serves on the speakers or advisory boards for GlaxoSmithKline and AstraZeneca.”
**Inadequate disclosure**		
“[Author] … has been paid to speak at a number of CME events as well as non-CME events.”	Trial of use of drug	“None of the [author group] have any significant financial involvement in any organization with a direct commercial interest in the subject discussed in the manuscript.”
“[Author] was paid $134,000 by AstraZeneca to assist in the marketing of Seroquel to pediatric patients.”	Trial of use of drug	“This research study was supported, in part, by NIMH grants MH58170 and MH56352 [to other author], and MH63373 ([author]).”
“AstraZeneca retained [author] to do numerous off-label talks and discussions on CME satellite and on-line programs…In 2003, AstraZeneca paid [author] $285,000 in return for his presentations to physicians.”	Review article in same field	“A roundtable for the authors in preparation for this supplement … was supported by AstraZeneca Pharmaceuticals LP.”

Table 2 shows how the adequacy of disclosure varied according to author and article characteristics in raw percentages, as well as the odds of adequate disclosure in each of these categories after adjusting for correlation of articles from the same author and same journal. Psychiatrists had a higher rate of adequate disclosure (26%) than other specialties, although after adjustment, we found no significant differences among specialties in authors' adequacy of disclosure. Disclosure rates varied little across authorship positions, or by whether the publication discussed an off-label use. Articles published in journals in each of the three higher quartiles of Journal Impact Factors had consistently greater odds of adequate disclosure than articles published in the lowest Journal Impact Factor quartile, but this difference was not statistically significant. In the raw data, commentaries appear to have the best rates of disclosure, but after adjusting for confounding by multiple publications from the same author, we found that commentaries were significantly less likely have adequate disclosure compared to articles reporting studies or trials (adjusted odds ratio = 0.10; 95% confidence interval = 0.02–0.67; p = 0.02).

**Table 2:** Association between adequacy of disclosure and characteristics of articles and authors.

Characteristic	Articles with Adequate Disclosure (N = 62)	Articles with Inadequate Disclosure (N = 342)	% with Adequate Disclosure	Adjusted odds ratio (95% CI)^a^	P value
**Author specialty (# of authors)**					
Psychiatry (13)	52	150	26%	[Ref]	[Ref]
Nephrology (9)	4	49	8%	0.65 (0.006–72.5)	0.86
Surgery/Urology (7)	4	107	4%	0.07 (0.001–5.86)	0.24
Neurology (5)	1	13	7%	2.72 (0–20.550)	0.83
Other (5)	1	23	4%	0.005 (0–1.693)	0.42
**Author's placement**					
Only	10	52	16%	0.50 (0.07–3.69)	0.50
First	18	69	21%	1.9 (0.26–14.02)	0.53
Middle	19	141	12%	0.87 (0.11–6.75)	0.90
Last	15	80	16%	[Ref]	[Ref]
**Type of article**					
Studies/trials	32	226	12%	[Ref]	[Ref]
Reviews	20	77	21%	0.38 (0.05–2.82)	0.35
Commentaries	10	39	20%	0.10 (0.02–0.67)	0.02
**Mentions off-label use**					
No	37	190	16%	[Ref]	[Ref]
Yes	25	152	14%	0.53 (0.15–1.83)	0.53
**Journal Impact Factor (quartiles)** b					
Lowest (<2.04)	9	90	9%	[Ref]	[Ref]
Second (2.04–3.61)	16	86	16%	1.61 (0.62–4.22)	0.33
Third (3.61–4.81)	21	75	22%	1.81 (0.71–4.63)	0.21
Highest (>4.81)	16	82	16%	1.55 (0.59–4.02)	0.37
**Article citation index per year since publication (quartiles)**					
Lowest (<1.0)	16	86	16%	[Ref]	[Ref]
Second (1–2.5)	17	87	16%	2.34 (0.60–9.08)	0.22
Third (2.5–5.5)	18	81	18%	1.92 (0.54–6.91)	0.32
Highest (>5.5)	11	88	11%	1.66 (0.35–7.93)	0.52

CI = confidence interval; Ref = reference group.

a Odds ratio adjusted for correlation of articles from the same author and same journal.

b Data missing for nine articles.

**Figure 2:**
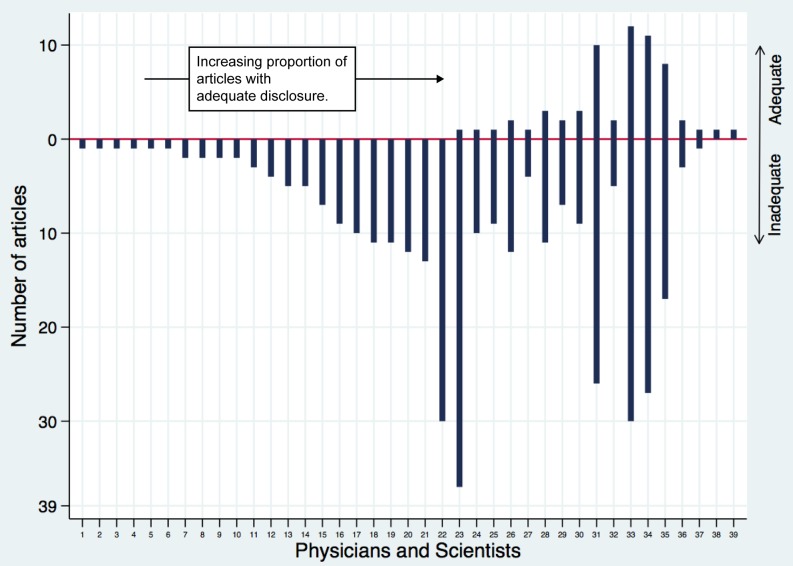
Counts and proportions of articles with adequate disclosure, by author. Each vertical line is a unique author, and the y-axis shows the number of articles published by that author. The extent of the vertical line above or below zero represents the frequency of adequate and inadequate disclosure for each author.

Aggregating articles by author provides a different perspective on the adequacy of disclosure (Figure 2). More than half (22/39, 56%) of the authors did not make an adequate disclosure in any publication. All but three authors (36/39, 92%) had inadequate disclosures in a majority of their publications, although there was considerable heterogeneity in the disclosure behavior of the most prolific authors. For example, among the 20 authors with more than five publications, more than half (12/20, 60%) made an adequate disclosure in at least one article. Among the six authors with 25 or more articles, two authors never or nearly never disclosed, but the other four disclosed in about one-third of their articles (range: 10/36 [28%] to 8/25 [32%]).

### Sensitivity Analyses

Restricting the analysis to publications that appeared 6–18 months after the latest date of the relationship reported in the complaint (as opposed to 6–36 months) reduced the count of related articles to 176, but did not change substantially the rates reported in Table 2 (unpublished data). Our companion analysis identified zero disclosures (out of 124) among unrelated articles.

## Discussion

In this study, we focused on whistleblower complaints, the only publicly accessible data that reveal the details of off-label marketing arrangements between pharmaceutical manufacturers and physicians. All of the relationships we identified were alleged by whistleblowers with special knowledge of company practices, although none of the complaints were subject to full trial and evaluation by a judge or jury. We found that, of 91 authors who had financial relationships with pharmaceutical companies in the context of off-label drug marketing, 39 authored 404 related articles in the three years following their engagement. However, only two-thirds of those articles contained any type of disclosure statement, one-quarter contained a disclosure statement that mentioned the relevant pharmaceutical company, and one in seven made disclosures that adequately described their relationship with the manufacturer. Adequate disclosure was no more or less likely in articles that discussed off-label uses. A majority of the most productive authors made adequate disclosures some of the time.

The rate of adequate disclosure we observed is markedly lower than rates detected in previous studies. For example, Okike et al. examined orthopedic surgeons with financial ties to hip and knee prosthesis manufacturers who presented or served as a committee or board member at an annual professional meeting, and found that 75% disclosed payments directly or indirectly related to their research (80% for the 208 directly related presentations, 50% for the 32 indirectly related presentations) [9]. Chimonas et al. examined publications from a subset of these orthopedic surgeons and identified disclosures in 50% of directly related and indirectly related articles published in the year following the payments (50% of the 52 directly related articles, 50% of the 34 indirectly related articles) [8]. The much lower rate of disclosure we observed may be due to consideration of authors from a range of clinical specialties, or our focus on ties with the pharmaceutical industry. Additionally, part of the difference may be attributable to the targeted nature of our study: authors who are paid by industry to support off-label uses may be especially poor disclosers.

Where does responsibility for this alarmingly high rate of inadequate disclosure lie? These failures spanned many articles and publications, pointing to authors themselves. Authors may be ignorant about what is required, may misunderstand the relatedness of the paper to their financial entanglement, or may be forgetful. The systematic nature of the non-disclosure, and context in which these failures occurred—suspect marketing activities that authors were paid to be a part of—suggest that embarrassment or willful hiding may explain at least some of the missing disclosures. However, this hypothesis is contradicted by the fact that physicians who are involved in off-label marketing activities tend not to face punishment by the DOJ or state medical boards [15], or to view their participation as inappropriate [30].

It is clear that that journal practices play a role in inadequate disclosure, because we found that some authors, including the most prolific ones, made adequate disclosures in some articles but not in others. It is unlikely that so many authors would engage in this type of behavior on their own, since it creates a public record of spotty disclosure that could be ascertained by searching and cross-referencing the medical literature [31]. To systematize practices, the ICMJE promulgated disclosure standards in 2009, including a template that requires authors to disclose direct support for the research, personal financial relationships, and other interests [32]. Yet journals still diverge in their disclosure requirements, and even in how they define a “conflict of interest” [33]–[34]. Journals also may inconsistently apply disclosure requirements to non-data-driven commentaries, as compared to reports of studies or trials. Indeed, our results reinforce other research that has found low rates of adequate disclosure among commentaries [35]. Our results, collected before the ICMJE standards were published, show some of the consequences of variable oversight by editors of biomedical journals.

Solutions to inadequate conflict of interest disclosure are not straightforward. Some have called for civil liability [36], and a few journals have threatened restriction of future publication [34]. Academic medical centers and universities have been a primary locus of attempts at reform, with many developing conflict of interest rules of varying intensity [37]. The Department of Health and Human Services has proposed requiring all universities and medical schools to disclose financial arrangements that could influence the work of government-funded researchers on their faculties [38], although this policy appears unlikely to be fully implemented [39].

While efforts by academic medical centers may enhance disclosure by authors of medical journal articles, we found that 57% of the physicians in the whistleblower complaints published no articles during the study period. Thus, manufacturers' off-label marketing strategies may often involve payments to physicians who have influence in their local communities, rather than those who engage in research or write articles related to practice. It is also notable that non-authors were more often provided with gifts or honoraria, while authors were more often paid as speakers on behalf of the company. The different types of financial inducements may reflect varying marketing roles played by physicians who contribute to the medical literature and those who do not. If academic medical centers tighten their policies about receipt of payments from manufacturers, more companies considering an off-label marketing strategy may continue to seek out such non-academically affiliated experts. The Patient Protection and Affordable Care Act will require manufacturers to report their physician payments to the government starting in 2013. This may promote transparency of financial relationships among all physicians, but its effectiveness will depend on the government's ability to make disclosures available in a timely and user-friendly fashion, and to prevent “laundering” of funds through seemingly neutral third-party corporations.

Our study has certain limitations. Our analysis is based on authors identified from whistleblower cases concerning off-label drug promotion. Rates and patterns of disclosure in this population may spotlight a “worst” end of the spectrum. Nonetheless, these authors are still capable of influencing prescribing practices, and the citation rate of their articles and the prestige of some of the journals in which their work was published deepen concerns that they have done so. In addition, while our results suggest deficiencies at both the author and journal level, our data cannot precisely define the fraction of deficiencies attributable to improper reporting by the author, as compared to administrative error, policies, or other reasons arising from the journal. This can be attributed in part to the fact that we did not obtain information on the disclosure requirements of each journal in the year of publication, nor did we have access to the specific disclosure-related communications between the journals and the authors. Finally, nearly all of the whistleblower complaints were focused on making out a case of fraud by the companies, not alleging or proving illegal activities on the part of the individual physicians or scientists that formed the focus of our study. Thus, the full range of payments to and interactions with individual physicians and scientists may not have been disclosed, rendering our account of these payments and interactions a lower bound on their true extent.

This study documented substantial deficiencies in the adequacy of conflict-of-interest disclosures made by authors who had been paid by pharmaceutical manufacturers. That such failures occurred in relation to off-label marketing schemes is especially troubling. Because off-label use is an area of clinical practice in which opinion is likely to be divided about appropriate care, the views of high-profile “opinion leaders” may exert considerable influence on prescribing practices [40],[41]. Disclosure of financial ties in these situations would give readers an opportunity to weigh the potential for bias. Our findings suggest that approaches to controlling the effects of conflicts of interest that rely on author candidness and variable policing by journals have fallen short of the mark. Readers are left with little choice but to be skeptical.

## Abbreviations

DOJ: US Department of Justice
FCA: False Claims Act
FDA: US Food and Drug Administration
IQR: interquartile range
OR: odds ratio
SE: standard error
